# Bilateral Vestibular Weakness

**DOI:** 10.3389/fneur.2018.00344

**Published:** 2018-05-31

**Authors:** Timothy C. Hain, Marcello Cherchi, Dario Andres Yacovino

**Affiliations:** ^1^Department of Otolaryngology, Northwestern University, Chicago, IL, United States; ^2^Department of Physical Therapy and Human Movement Science, Northwestern University, Chicago, IL, United States; ^3^Department of Neurology, Northwestern University, Chicago, IL, United States; ^4^Department of Neurology, Dr. Cesar Milstein Hospital, Buenos Aires, Argentina

**Keywords:** bilateral vestibular weakness, oscillopsia, ototoxicity, vestibulo-ocular reflex, rotatory chair testing, vestibular testing

## Abstract

Bilateral vestibular weakness (BVW) is a rare cause of imbalance. Patients with BVW complain of oscillopsia. In approximately half of the patients with BVW, the cause remains undetermined; in the remainder, the most common etiology by far is gentamicin ototoxicity, followed by much rarer entities such as autoimmune inner ear disease, meningitis, bilateral Ménière’s disease, bilateral vestibular neuritis, and bilateral vestibular schwannomas. While a number of bedside tests may raise the suspicion of BVW, the diagnosis should be confirmed by rotatory chair testing. Treatment of BVW is largely supportive. Medications with the unintended effect of vestibular suppression should be avoided.

## Introduction

Reduced or absent vestibular function on both sides, resulting from deficits in the labyrinths, or vestibular nerves, or their combination, is referred to in the recent consensus statement from the Bárány Society (1) as “bilateral vestibulopathy.” Although much of the literature designates this phenomenon “bilateral vestibular *loss*,” that phrase is inappropriate when the deficit is partial rather than complete. In this review, we prefer the more neutral designation *bilateral vestibular weakness* (BVW).

We discussed this topic in 2013 (2), but a considerable number of publications since that time warrant inspection in the context of a broader review. Here, we discuss additional etiologies of BVW, we reassess the category of “idiopathic” cases, and we review the relevance of emerging diagnostic technologies for this disease.

Bilateral vestibular weakness can involve different combinations of labyrinthine components. For example, gentamicin ototoxicity affects the entire labyrinth (with variable degrees of severity), whereas bilateral sequential vestibular neuritis tends to involve the superior divisions of the vestibular nerves (see discussion below).

In this review, we will use terms such as mild, moderate, and severe BVW, recognizing that there are currently no generally accepted quantitative criteria associated with these designations.

## Clinical Features and Symptoms of BVW

### Oscillopsia

Bilateral vestibular weakness almost invariably produces the symptom of oscillopsia—the illusion that the environment moves when the head does. Oscillopsia is due to malfunction of the vestibulo-ocular reflex (VOR), is nearly always due to a peripheral vestibular deficit, and is only rarely due to a central (e.g., brainstem) vestibular deficit. Oscillopsia can occur even with small, “natural” head movements, such as when walking. During ambulation there is rhythmic, modest flexion-extension of the neck in the sagittal plane with each step; in a healthy person the VOR ensures that such head movement is exactly offset by equal but opposite movement of the eyes, such that the seen world appears stationary to the individual. In BVW, the VOR fails to drive this compensatory eye movement adequately, so the individual will perceive the seen world as swaying or bouncing with each step. Similar disturbances occur with abrupt passive movements, such as when riding in an automobile on a bumpy road (3). The heavily “visual” nature of the symptom of oscillopsia often misleads patients into thinking that their imbalance arises from a primary ophthalmological disorder.

### Imbalance

Patients with BVW almost always complain of imbalance. This symptom is sensitive, though not specific for BVW. In order to determine one’s position, orientation in and movement through space, the brain draws on three main sensory modalities (visual, proprioceptive, and vestibular input) and on internally generated estimates (derived from differences between those sensory inputs and motor efference copies). In a patient with BVW, the brain will try to compensate for the reduced vestibular input by relying more heavily on the unaffected sensory modalities (visual and proprioceptive) and on internal estimates. If the previously unaffected sensory inputs are impaired, then the symptom of imbalance will worsen. For example, if vision is impaired abruptly (such as by trying to walk in a poorly illuminated area) or gradually (such as from cataracts or macular degeneration), or if proprioception is challenged abruptly (such as when walking on a soft or uneven surface) or deteriorates gradually (such as with diabetic peripheral neuropathy), then the balance in a patient with BVW will suffer.

### Auditory Symptoms

Auditory symptoms such as hearing loss and tinnitus are not common features of BVW. One plausible reason for this is that the common cause of BVW, gentamicin ototoxicity, is predominantly vestibulotoxic rather than cochleotoxic. Even in cases of BVW of undetermined etiology, auditory symptoms are uncommon. Of the uncommon causes of BVW, etiologies that damage the entire inner ear (such as meningitis or congenital labyrinthine hypoplasia) cause both vestibular and auditory symptoms.

### Epidemiology

The prevalence of BVW is low. A rough estimate of prevalence was provided by Ward et al. (4), who surveyed more than 21,000 adults for symptoms of oscillopsia and ataxia, lasting at least 1 year, with symptoms being “a big problem.” They estimated that the prevalence of BVW is 28/100,000. While a valuable step forward, this estimate obviously has a rather wide error margin, as there were no vestibular measurements made.

Another method of estimating prevalence is determining its relative frequency of diagnosis. Of cases accrued in our clinical practice over 20 years, 213 patients (out of a total of approximately 25,000) were diagnosed with BVW, amounting to approximately 0.7%. So, whether one considers prevalence in the population, or frequency of presentation in a “dizzy” clinic, BVW is rare.

### Etiologies

The etiologies of BVW are usually listed as including ototoxicity, autoimmune inner ear disease (AIED), bilateral versions of what are more commonly unilateral diseases (e.g., vestibular neuritis, Ménière’s disease, and tumors), with the remainder designated “undetermined” or “idiopathic” (5). One series of 53 cases (6) reported that 39% were associated with neurological disorders (13% cerebellar degeneration, 11% meningitis, and 9% had an association with cranial or peripheral neuropathies), 21% were “idiopathic,” 17% were due to gentamicin ototoxicity, 10% were due to autoimmune disease, 8% were attributed to bilateral occurrence of what would usually be unilateral disease (e.g., temporal bone fracture, Ménière’s disease), and 6% were associated with tumors.

Other case series of BVW patients (6–8) reported “idiopathic” or “unknown” to be the largest single subcategory, aminoglycoside ototoxicity the second most common, and infections (such as vestibular neuritis or meningitis) the third most common.

Familial BVW, with or without hearing loss, is rare and has been reviewed elsewhere (9). Another possibly genetic syndrome is cerebellar ataxia, neuropathy and vestibular areflexia syndrome (CANVAS), in which the “vestibular areflexia” refers to bilateral vestibular weakness (10); this is exceedingly rare.

**Figure 1:**
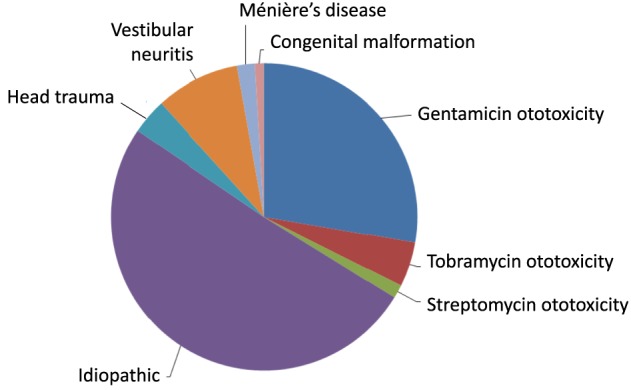
Distribution of etiologies of bilateral vestibular weakness at Chicago Dizziness and Hearing, *n* = 213.

The causation of BVW is usually estimated from clinical data. The distribution of BVW cases accrued in our practice in Chicago, IL, is displayed in Figure 1. Of 213 patients with bilateral weakness diagnosed on rotatory chair testing (RCT) (which, as we shall discuss below, is regarded as the gold standard for assessing BVW), the most common etiologies, in order of descending frequency, were “idiopathic” (50.7%), followed by gentamicin ototoxicity (27.7%), bilateral vestibular neuritis (8.9%), tobramycin ototoxicity (4.7%), head injury (3.8%), autoimmune (3.3%), Ménière’s disease (1.9%), streptomycin ototoxicity (1.4%), and congenital (0.9%).

### Age

The median age in our series of patients with BVW was 56 years. While this may be due in part to the age distribution of the general population in Chicago, it more likely reflects that BVW tends to be a disease of older age. As one grows older, there is simply more opportunity to suffer ear damage, such as from vestibular neuritis or ototoxicity.

The prevalence of balance problems increases with age (11–13). Histopathological studies of the temporal bones of otherwise healthy individuals demonstrate a steady decline of vestibular hair cells over time (14). Studies of the vestibular nerve show that by the age of 80, the number of fibers in the vestibular nerve declines by about 30–50% (15–17). Despite this loss, the evidence for age-related loss of semicircular canal function is not compelling; for instance, a 30–50% deficit in semicircular canal function in otherwise healthy individuals does not appear to increase the risk of falls significantly (18).

In contrast, there is far stronger evidence for age-related loss of otolith function than loss of semicircular canal function, as clinical tests of otolith function such as vestibular-evoked myogenic potentials (VEMPs) are generally greatly diminished with age (19). Thus, at this writing, it seems likely that while imbalance may be fairly strongly correlated with age and VEMP amplitude, it is only weakly attributable to loss of the canal mediated VOR. When attrition of labyrinthine function is combined with decline of other sensory inputs (such as visual or proprioceptive loss), vestibular symptoms are magnified.

## Ototoxic Causes of BVW

### Aminoglycoside Antibiotics

After idiopathic sources are excluded, the aminoglycoside antibiotics, gentamicin, and tobramycin, are the single most common source of severe BVW.

All aminoglycoside antibiotics are potentially ototoxic, though some (such as gentamicin and streptomycin) are predominantly vestibulotoxic (20), while others (e.g., neomycin) are preferentially cochleotoxic.

Of the vestibulotoxic agents, gentamicin is the most frequently encountered in vestibular clinics. Tobramycin (which is both vestibulotoxic and cochleotoxic) is the second most common because of its use in the treatment of cystic fibrosis; its risk of ototoxicity is relatively higher when administered intravenously (21–27), and is very low when inhaled (28–31). Streptomycin is rarely used anymore in the United States, and consequently it is seldom the cause of BVW.

The high prevalence of gentamicin ototoxicity is due to several features of its pharmacology. First, gentamicin does not produce auditory “warning signs” (hearing loss or tinnitus) that would alert a patient or treating physician to impending toxicity. Second, even though most aminoglycosides (including gentamicin) are renally excreted within hours, gentamicin accumulates over months in the inner ear (32), and it is this accumulation that accounts for the drug’s ototoxic effects even in patients whose serum concentration has remained within normal limits over the course of treatment. Third, gentamicin is both ototoxic and nephrotoxic; as renal function declines and gentamicin excretion decreases, the drug level (and its ototoxic and nephrotoxic effects) escalates, resulting in a positive feedback loop of toxicity. Fourth, gentamicin’s ototoxicity is potentiated by vancomycin (33), which is commonly administered simultaneously.

In some individuals, particular susceptibility to gentamicin ototoxicity appears to be due to genetic factors (NOS3, GSTZ1, and GSTP1) (34). Finally, gentamicin is inexpensive and readily available, which may promote its use.

Aminoglycoside ototoxicity usually occurs in the context of intravenous or (less commonly) intraperitoneal administration. However, if aminoglycoside-containing agents are instilled directly into the middle ear (such as through a tympanic membrane perforation), they can diffuse through the round window membrane to the inner ear and cause damage. For this reason, when considering direct aural administration of aminoglycoside-containing agents such as Cortisporin Otic^®^ (which contains neomycin), or gentamicin ophthalmic solution (used off-label), one should ensure that no tympanic membrane perforation is present (35–37).

### Chemotherapeutic Agents

Several chemotherapeutic agents have cochleotoxic potential. Only cisplatin is clearly vestibulotoxic (38), yet this is rarely seen, probably because the drug’s other toxicities limit its use before vestibulotoxicity becomes manifest.

### Other Medications

There are scattered reports of various medications appearing to cause BVW, though isolated case reports comprise weak evidence. The evidence for some of these appears stronger, such as a series describing 15 out of 126 patients (12%) with what otherwise appeared to be “idiopathic” BVW who had been treated with amiodarone (39).

## Non-Ototoxic Causes of BVW

### Autoimmune Inner Ear Disease

Autoimmune inner ear disease and its subtypes are rare causes of BVW. AIED tends to affect both auditory and vestibular function. This condition generally presents with bilateral sensorineural hearing loss that progresses over weeks to months, and the diagnosis is confirmed when this hearing loss improves significantly (or resolves) after a brief course of high-dose steroids (40, 41). Diagnosis of AIED by antibody-based assays (e.g., HSP-70) has proven unreliable (42). Although a steroid burst can improve the hearing loss in AIED, the high doses that are required generally preclude their long-term use. Long-term pharmacologic management can be attempted with TNF-alpha blockers [e.g., etanercept (43, 44), adalimumab (45), or possibly rituximab (45, 46)]. If that fails, then cochlear implantation can be considered—though obviously this does not address BVW, if present. Approximately half of cases of AIED also involve vestibular symptoms (47). There are case reports of AIED presenting exclusively with vestibular symptoms and no hearing loss (48), but it is unclear how one could be confident in the diagnosis if there is no opportunity to assess for steroid-responsive hearing loss.

There are a few other inner ear conditions that appear to be immunologically mediated. First, patients who have undergone inner ear surgery on one side may develop auditory and vestibular symptoms in the opposite (un-operated) ear; this condition is thought to be a “sympathetic autoimmune reaction,” analogous to the ocular involvement of Vogt–Koyanagi–Harada syndrome of the eye (49). Second, Cogan syndrome (50) is similar to AIED but additionally has ocular symptoms; in some respects it resembles post-meningitic hearing loss (see below), as the labyrinth may be occluded with fibrous tissue.

### Meningitis

**Figure 2:**
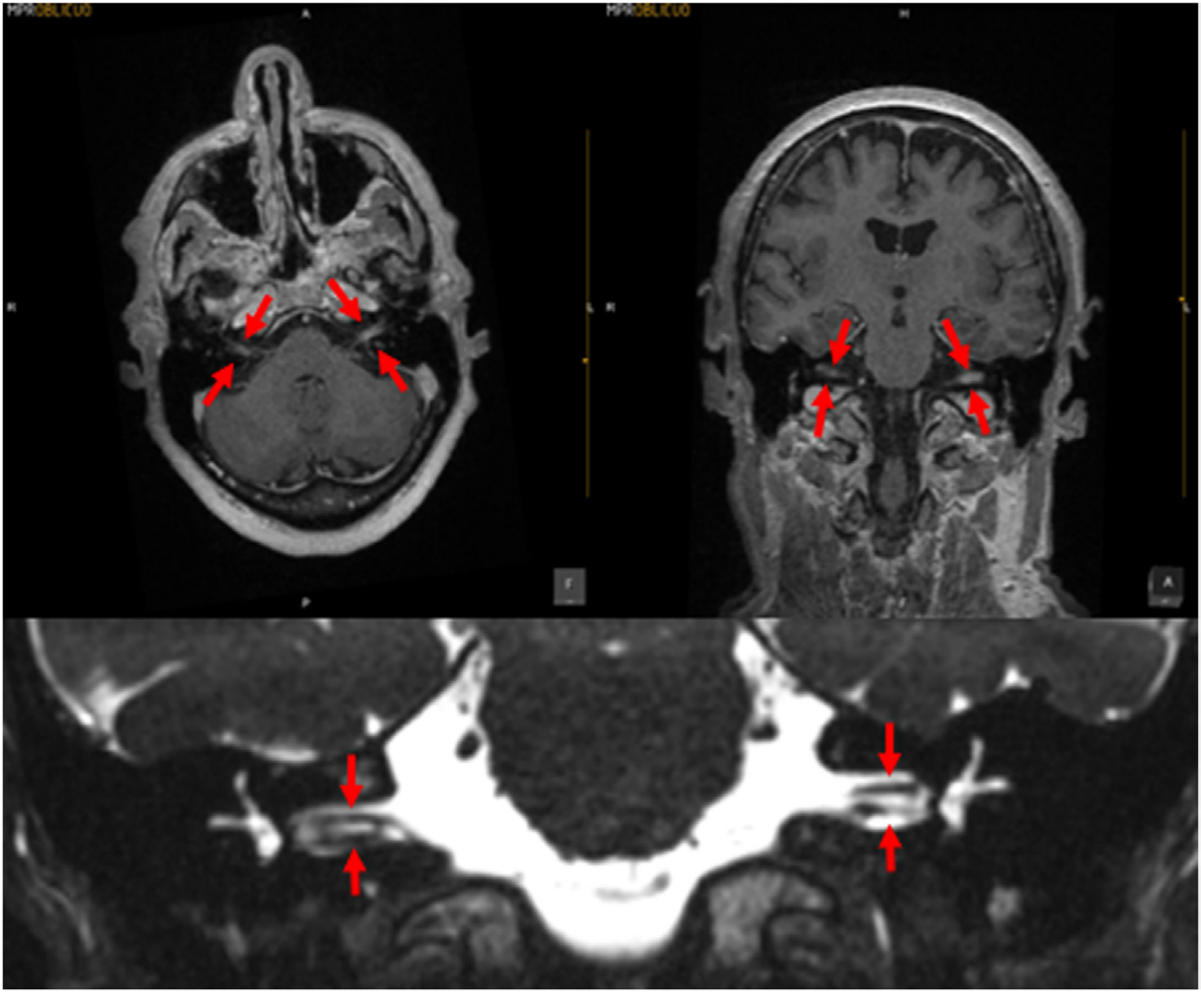
MRI of a patient with meningitis. The upper left panel is a post-contrast T1 axial image; the upper right panel is a post-contrast coronal image; the arrows indicate enhancement of the vestibulo-cochlear nerves. The lower panel displays a coronal CISS sequence image; the structures indicated by the arrows demonstrate that the vestibulocochlear nerves are of relatively normal caliber, with no evidence of vestibular schwannoma. Images courtesy of Dr. Manuel Perez Akly.

Meningitis can damage the entire labyrinth (51), but tends to affect cochlear function more than vestibular function (52). The meningitic inflammation likely reaches the ear through the vestibular and cochlear aqueducts (53); these passages are more patent during childhood, which may be the reason that children are more likely than adults to develop hearing loss following meningitis (54). In many cases of meningitis, the hearing loss and vestibular deficits manifest immediately, while in other cases the vestibular weakness may develop more gradually; such delay is often attributed to the slow development of fibrosis or ossification of the inner ear, which can sometimes be visualized on high-resolution MRI. In Figure 2 is displayed a brain MRI in a patient with meningitis, showing enhancement in both internal auditory canals.

### Bilateral Vestibular Neuritis

Vestibular neuritis can affect any combination of afferent fibers (55), and thus can involve the superior or inferior divisions, or both. Perhaps due to anatomical factors (56, 57), vestibular neuritis more commonly involves only the superior division of the vesbitular nerve (55, 58), less commonly involves both the superior and inferior divisions (55), and uncommonly affects only the inferior division (59–61). Given this pattern, it is unsurprising that when vestibular neuritis is bilateral, it tends to involve the superior division on both sides (62, 63). It is possible, though uncommon, for bilateral sequential vestibular neuritis to involve the superior division on one side and the inferior division on the other (64). Rare cases of bilateral inferior division deficits [identified on cervical VEMPs (cVEMPs)] have been reported (65), but it is unclear whether these are due to bilateral vestibular neuritis. The tendency of vestibular neuritis to involve the superior division of the vestibular nerve can have diagnostic value in bilateral cases; for instance, if a patient has evidence of bilateral superior division weakness [on caloric testing, RCT, or video head impulse testing (vHIT)] but preserved inferior division function (with intact cVEMPs), then this pattern is more likely to be due to bilateral vestibular neuritis (rather than due to processes that involve the entire labyrinth or the entire vestibular nerve). Loss of caloric function, by itself, is insufficiently specific as caloric testing assesses the horizontal canal alone.

### Bilateral Vestibular Schwannomas

Neurofibromatosis type 2 can manifest with bilateral vestibular schwannomas (66) resulting in BVW. This is exceedingly rare.

### Bilateral Ménière’s Disease

The most notable features in the typical clinical history of Ménière’s disease are the dramatic episodes of vertigo and accompanying auditory symptoms, but the overall trajectory—typically over years to decades (67)—is one of gradually progressive sensorineural hearing loss. However, it is rare for the disease to progress to profound deafness. It is similarly rare to develop the vestibular analog—severe vestibular weakness. In cases of bilateral Ménière’s disease (68), hearing usually remains in the aidable range, and patients generally do not develop severe BVW.

### Neurosyphilis

When neurosyphilis involves the ear, the usual presentation is hearing loss. Vestibular manifestations are reported in 42% (69) to 52% (70) of cases. Some series report that 80% of patients with vestibular symptoms have electronystagmographic abnormalities (71), and some of these are BVW (70, 72). The widespread use of antibiotics has dramatically reduced the prevalence of neurosyphilis, so testing for this is low yield. Nevertheless, some clinicians advocate checking for this routinely, as it is one of the few potentially treatable causes of BVW.

### Superficial Siderosis

**Figure 3:**
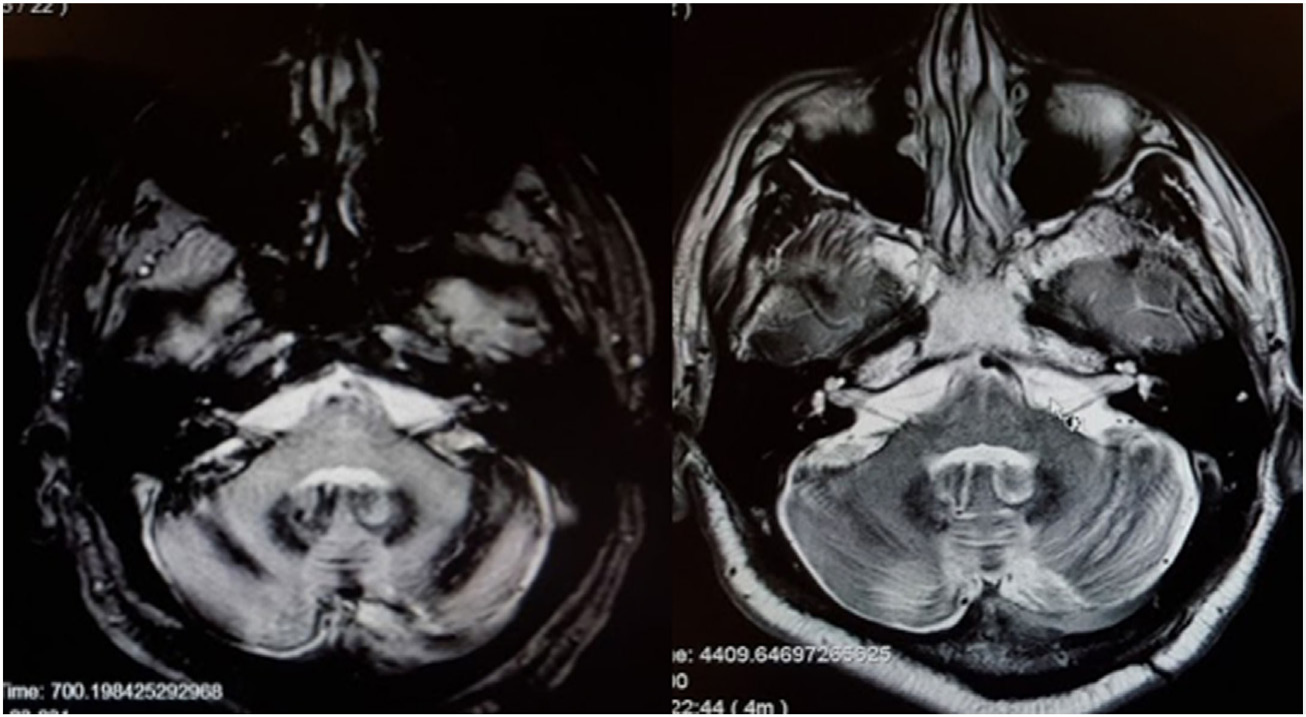
MRI of a patient with siderosis showing hemosiderin deposition along the vestibulocochlear nerves. The image on the left is an axial gradient-echo (GRE) T2*-weighted sequence. The image on the right is an axial T2-weighted image. Both figures are through the internal auditory canals. The study was performed on a 1.5-Tesla strength MR.

There are scattered case reports of superficial siderosis resulting in BVW (73, 74). It is usually suggested that the pathological process involves deposition of hemosiderin along the glial segment of the vestibulocochlear nerve (75–77) rather than direct damage of the labyrinth. Superficial siderosis can damage auditory function, vestibular function, or both. In our clinical practice, we encountered a patient with total deafness due to superficial siderosis who had preserved vestibular function. We have also encountered a patient (MRI displayed in Figure 3) with both hearing loss and BVW.

### Vascular Causes

Bilateral vestibular weakness seldom results from focal circulatory disturbances. Vascular supply to the inner ear is *via* the labyrinthine artery (generally a branch of the anterior inferior cerebellar artery). Unilateral labyrinthine infarction is very rare (78); in order for bilateral labyrinthine infarction to occur, both labyrinthine arteries or AICAs would need to be compromised, which is a statistically extremely unlikely event. If BVW arises from a circulatory disturbance, the etiology is more likely to be a more diffuse vasculopathic/vasculitis process; for instance, we have encountered BVW in one patient with granulomatosis with polyangiitis (Wegener’s granulomatosis); there are also several published cases of what appears to be BVW (based on RCT) in patients with Behçet’s disease (79).

### Neurosarcoidosis

Sarcoidosis has no particular propensity for the vestibular nerve or labyrinth. It is a very rare cause of unilateral ear damage (80), and thus a very implausible etiology of BVW.

### Congenital Malformations

**Figure 4:**
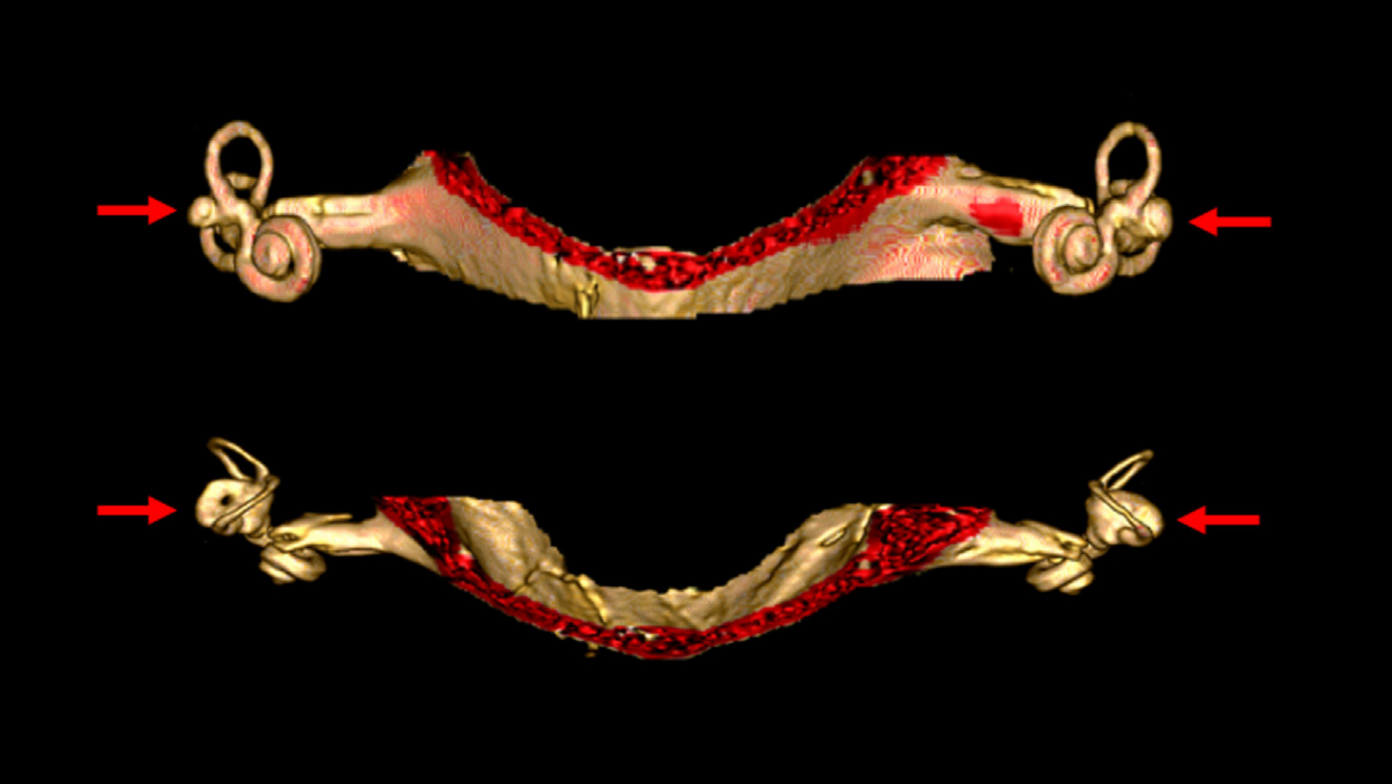
High resolution three-dimensional reconstruction MRI of the internal auditory canals and inner ear structures of a patient with bilateral vestibular weakness from bilateral labyrinthine dysplasia. The top image is in the coronal aspect. The bottom image is in the axial aspect. In these images it is evident that the horizontal canals are dysplastic, with the horizontal canal and vestibule appearing as a single abnormal structure on each side (indicated by the arrows). The superior and inferior canals are present as true canals, but are somewhat hypoplastic.

Malformations of the vestibular end organs occur in a number of congenital disorders (81), though very few such disorders have been sufficiently studied to ascertain whether they truly involve BVW. Aplasia of the semicircular canals (82) occurs in a few rare conditions such as coloboma of the eye, congenital heart defects, choanal atresia, mental and/or growth retardation, genital hypoplasia, ear anomalies and/or deafness and Mondini malformations. Imaging in these cases may demonstrate partial or total absence of the labyrinth; these patients have congenital deafness (83). Figure 4 shows labyrinthine hypoplasia in a patient with BVW.

### Head Trauma

If head injuries damage the inner ear, they generally do so *via* a labyrinthine concussion or a traction injury of the vestibulocochlear nerves (84). Typically, an injury sufficient to cause such damage will result in both vestibular and auditory deficits. The temporal bone is the hardest bone in the body; any impact that damages the labyrinth or vestibulocochlear nerve *via* a temporal bone fracture (85) will almost invariably result in brain injury as well.

## Associations of Other Conditions with BVW

### Migraine

Several case series (86, 87) report an association between migraine and BVW. However, since migraine is so extraordinarily common, it is difficult to ascertain whether this combination truly represents a distinct subtype of migraine (88), or whether it is merely chance overlap of two independently occurring conditions.

### Cerebellar Degeneration

The combination of cerebellar degeneration and BVW has been reported in several case series (6, 10, 89–91). Since both conditions can manifest with ataxia, a clinical examination can easily “catch” the cerebellar dysfunction but miss the BVW. The association of these two conditions with a third, peripheral neuropathy, has been designated CANVAS (10, 89, 90), as mentioned earlier. In our clinic, these patients comprise less than 2% of cases of BVW, and given their additional deficits (ataxia, peripheral neuropathy), their prognosis is poorer than those with BVW alone.

### Idiopathic

Although we have discussed the literature as it pertains to known causation of BVW, as we previously mentioned, most clinical series find “idiopathic” to be the most common “diagnosis.” In Figure 1 showing 213 patients found in our clinic setting, half were idiopathic.

It seems possible that at least some of these patients are actually individuals with bilateral vestibular neuritis, as vestibular neuritis is a relatively common inner ear condition, and the bilateral variant of it is well established (62, 63). Nevertheless, this would imply that the prevalence of vestibular neuritis is much higher than generally accepted. One can deduce this by using Ward’s prevalence figure of 28/100,000 (4) for BVW, and assuming all of the idiopathic cases are from bilateral vestibular neuritis, or 14/100,000, then the square root of this figure should be the prevalence of vestibular neuritis. This would imply a prevalence of about 1% for unilateral vestibular neuritis, which is much higher than the generally accepted figure.

## Diagnosis of BVW: Clinical Examination

Dynamic visual acuity (DVA) testing, sometimes also called dynamic illegible E testing (92, 93), can be helpful bedside examinations when considering a diagnosis of BVW. This test is performed by comparing visual acuity while the patient’s head is stationary, to that when the head is oscillated from side to side. Different methods have been described, but typically the passive sinusoidal rotation of the head is performed over an arc of 15–30° to each side, with a frequency of 1–2 Hz. Visual acuity in the stationary and oscillating conditions is assessed by having the patient read the smallest letters he or she can on an eye chart whose lines are arranged by descending LogMARs (logarithmic change in the minimum angle of resolution)—different from the organization of a Snellen chart. An example of a LogMAR-based eye chart is available on our website (94). Some authors suggest that a loss of more than two lines (0.2 LogMARs) should be interpreted as supporting a diagnosis of BVW, though in our experience some normal individuals can perform in this way. The requirement of a loss of four lines (0.4 LogMARs) is more specific. In patients with BVW from gentamicin ototoxicity, their performance on the DVA rarely improves to a difference less than 0.4 LogMARs.

The bedside HIT was originally recognized as a method for detecting unilateral vestibular weakness (95), but can also serve for detecting bilateral weakness. The underlying concept is similar to DVA, but the technique differs. Whereas the DVA depends on the patient’s report of what line on the LogMAR chart he or she is able to read, the bedside HIT instead depends on the examiner’s ability to observe a catch-up saccade following a high acceleration, low-amplitude rotation of the patient’s head while the patient is attempting to maintain his or her gaze fixed on a target; these compensatory saccades occurring after the head movement is completed are called “overt saccades.” The sensitivity and specificity of the bedside HIT depends on patient cooperation, as well as on the examiner’s skill (both in executing the maneuver and observing the compensatory saccades). Patients with cervical pain or limited cervical range of motion may not tolerate this test well. An additional problem is that as patients improve they may learn to produce the compensatory saccades during (rather than after) the head rotation; these are called “covert” saccades and are more difficult for the examiner to observe (96)—in other words, the sensitivity of this test may diminish over time. The problem of identifying covert saccades can be addressed by a computerized version of the HIT called vHIT (discussed below).

### Ophthalmoscope Test

The principle underlying this test is similar to DVA, but the technique is different. Whereas the DVA depends on the patient’s report of what line on the LogMAR chart he or she is able to read, the ophthalmoscope test instead depends on the examiner’s ability to observe (with an ophthalmoscope) the patient’s retina during passive oscillation of the patient’s head. Keeping the retina in view during movement of the patient’s head can be challenging, so the amplitude of oscillation should be 10–20°, and the frequency of oscillation should be approximately 1 Hz (97). During the passive oscillation of the head in a healthy person, the retina should appear (to the examiner) to remain still. In contrast, a BVW patient’s retina will appear to oscillate in synchrony with the head oscillation, because the vestibular system is unable to generate compensatory eye movements to offset the head movements. The ophthalmoscope test should be performed while the patient is wearing any corrective lenses that they usually wear for distance viewing. Similar to the bedside HIT, a patient’s performance on the ophthalmoscope test can improve over time (98). The ophthalmoscope test is highly specific, but it is not sensitive (99).

### General Comments About Bedside Testing for BVW

Dynamic visual acuity testing, bedside HIT, and the ophthalmoscope test are performed when vision is available, and with high acceleration, and are therefore termed “light, high-frequency tests.” However, BVW is more evident when visual fixation is unavailable, and when the head acceleration is smaller (i.e., in “dark, low-frequency” conditions), so the DVA, bedside HIT, and ophthalmoscope test are not nearly as sensitive as RCT (discussed below). If a there is a high index of suspicion for BVW but the DVA, bedside HIT and ophthalmoscope test are normal, then one should proceed to RCT.

## BVW: Vestibular Laboratory Testing

### Rotatory Chair

**Figure 5:**
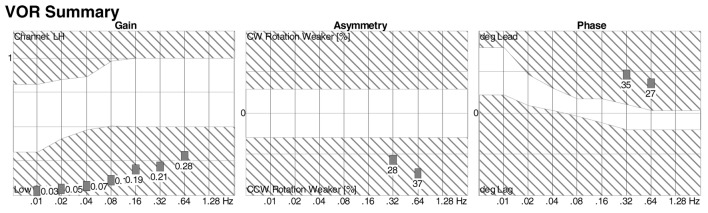
Slow harmonic acceleration from rotatory chair testing. The panel on the left is a plot of the gain, which is low at all frequencies. The panel on the right is a plot of the phase, which could not be calculated at 0.01–0.16 Hz, and showed phase lead at 0.32–0.64 Hz.

Rotatory chair testing is still regarded as the gold standard test for BVW (100, 101). This is due to the fact that it measures responses ranging from high to low frequencies (i.e., high to low acceleration), unlike the situation with the caloric test (see below) that assesses only the very low frequencies, and the vHIT test (see below) that assesses only the high frequencies. The responses to the range of stimulus frequencies are plotted during part of the RCT termed slow harmonic acceleration. The deficits in BVW are most evident in the lower frequencies, where one observes low gain and phase lead (if phase can be measured at all), as shown in Figure 5. The equipment required for performing RCT properly is expensive, so its availability is mostly limited to major academic medical centers. Consequently, there have been attempts to develop less expensive devices, such as the VORTEQ^®^ (“VOR test equipment”) (102, 103), but these apparatuses actually only provide “light, high-frequency” assessments and are thus no more sensitive than the bedside tests (DVA, HIT, and ophthalmoscope test) described above, so they cannot truly substitute for RCT. It should also be noted that tests (such as the VORTEQ^®^) in which the patient actively rotates his or her head (rather than having it passively oscillated by the examiner) reduce the sensitivity of the test, because active performance of head-on-neck movements also enables “pre-programming” of ocular movements that has been shown to augment the VOR (104).

### Videonystagmography

The caloric portion of videonystagmography (VNG) is sensitive to bilateral vestibular weakness. The thermal stimulus delivered by warm and cool water caloric testing is generally cited as equivalent to an oscillation frequency of 0.003 Hz (101, 105–107), thus it is a “low frequency test,” hence its sensitivity for bilateral vestibular loss (108). The total caloric response is the sum of two cool calorics (one in each ear) and two warm calorics (one in each ear). The average total caloric response in healthy individuals is usually cited as 100°/s (109). The threshold beneath which BVW can be diagnosed is debated; Zapala et al. (109) state that, “Fewer than one in 100 otherwise normal subjects demonstrates a T(otal) E(ye) S(peed) of less than 27°/s”; the Bárány Society Consensus document on BVW (1) states that, “the lower limit of the normative data… varies among laboratories from 20 to 25°/s,” yet lists the diagnostic criterion (for VNG) as “reduced caloric response [sum of bithermal max(imum) peak S(low) P(hase) V(elocity) on each side <6°/s],” implying that a total caloric response <12°/s is diagnostic. Even if one uses a very stringent criterion [such as ≤10°/s as the cutoff studied by Furman and Kamerer (100)], some individuals identified on caloric testing as having BVW nevertheless have normal responses on RCT (100, 110), showing that caloric testing can render falsely positive results. False positives may be a consequence of a number of factors, including the presence of cerumen, narrow ear canals, or the use of weak stimuli such as balloon irrigation or air caloric stimulation (111). Conversely, caloric testing can miss moderate BVW, and can thus also render falsely negative results. False negatives are probably due to the fairly wide range of normal responses in healthy controls. One difficulty in interpreting the results of caloric testing is that many laboratories do not report whether caloric testing was performed with air or water stimulation; air calorics comprise a weaker thermal stimulus and pose a greater risk of false positives. In the appropriate clinical context, if caloric testing reports BVW, this should be confirmed on RCT.

### Video Head Impulse Testing

**Figure 6:**
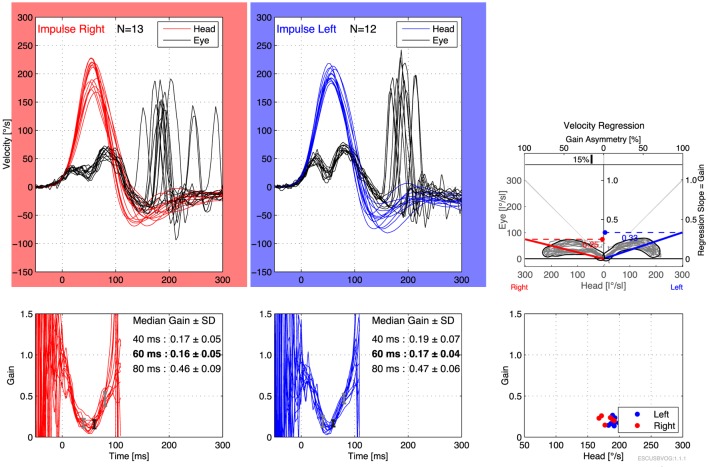
Video head impulse testing of the horizontal canals in bilateral vestibular weakness, showing bilaterally low gain and overt saccades.

**Figure 7:**
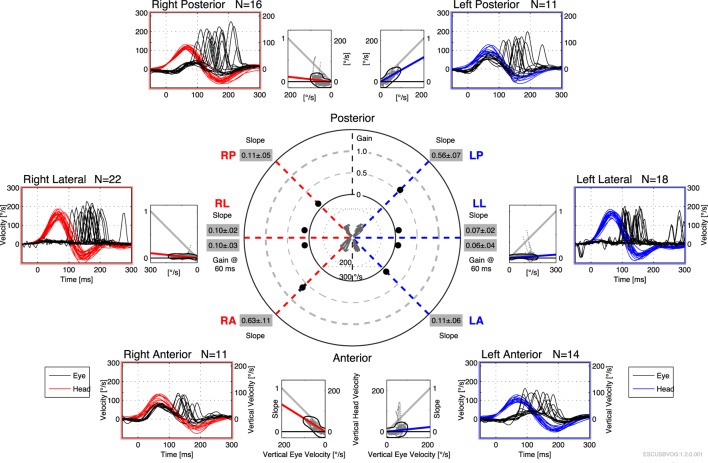
Video head impulse testing of all six semicircular canals in bilateral vestibular weakness, showing diffusely low gain and overt saccades.

The first reports of the clinical utility of the bedside version of the HIT emerged in the 1980s (95), but it was recognized that the maneuver can be difficult to execute and the observation of the elicited saccades can be difficult. Technology has been developed to address this in the form of vHIT. The early versions of this technology were custom designed and restricted to research settings (112–115), but the technology has evolved and is now more readily available and affordable. Commercially available products both monitor the movement of the head during the impulse (to ensure that head acceleration is adequate) and process the video of the elicited eye movement (to characterize the resulting compensatory saccades), making it easier to recognize corrective saccades and quantifying the gain, as shown in Figures 6 and 7. It appears that vHIT is superior to bedside HIT (116). There is a modest literature (73, 75, 115, 117) suggesting that vHIT can play a valuable role in the identification of BVW, but its sensitivity and specificity (compared with the gold standard of RCT) has yet to be established. Comparison of vHIT with RCT is complicated by several factors that can introduce variability into calculation of VOR gain on vHIT; the first reason is physiologic, and has to do with the fact that target viewing distance (which may not be carefully controlled) can have a significant effect on gain both in healthy individuals (118–120) and in patients with peripheral vestibular disease (121); the second reason is technological, as it has been demonstrated that different devices and different algorithms can render different results in gain (122).

Advantages of the vHIT include that it can be performed rapidly, the equipment is far less expensive than the rotatory chair device, it can assess all six semicircular canals (as shown in Figure 7) and the vHIT is relatively difficult to affect with use of medication or lack of patient cooperation. Nevertheless, the vHIT test is not as capable as the rotatory chair as it is not designed to detect low-frequency vestibular responses. The predominant frequency of stimulation in the vHIT is 2.5 Hz (123). In our opinion, using the vHIT as one’s sole vestibular test is similar to testing the hearing at 4 kHz, and suggesting that this is a proxy for hearing at all frequencies. In our clinical practice, we view the vHIT as a convenient screening test, but still rely on RCT for confirmation of BVW.

### Cervical Vestibular-Evoked Myogenic Potentials

In clinical practice, the VEMP response is most commonly measured from the sternocleidomastoid muscle and is usually designated a cVEMP. The response is believed to be mediated by the saccule and its afferents through the inferior division of the vestibular nerve (124). The presence of conductive hearing loss makes sound-conducted VEMPs non-diagnostic, so in this circumstance the stimulus must be delivered by bone vibration. All VEMPs are also known to decline significantly with age (19), so their diagnostic utility diminishes in the elderly. In a young or middle age person with no conductive hearing loss, cVEMPs can help distinguish whether BVW is due to a condition affecting the entire inner ear (such as gentamicin ototoxicity or meningitis, in which the cVEMP should be reduced or absent) or due to a condition with an incomplete lesion (such as bilateral sequential vestibular neuritis, which more commonly involves the superior division of the vestibular nerve and thus will have preserved cVEMPs).

A related evoked potential test, ocular VEMPs (oVEMPs), has been developed more recently (125, 126) and is believed to evaluate the function of the utricle and its afferents through the superior division of the vestibular nerve (127, 128), but has not yet been studied well in the population of patients with BVW. As oVEMP amplitudes decline precipitously with age, and bilateral loss tends to affect an older population, one would expect that oVEMPs would be far less useful than tests that depend on semicircular canal function, as canal function is little affected by age (129).

### Computerized Dynamic Posturography (CDP)

Computerized dynamic posturography, while sensitive for BVW, is not specific insofar as it fails to distinguish BVW from several important and more common neurological causes of imbalance [e.g., ataxia from cerebellar lesions (130)]. On CDP, BVW patients will generally exhibit a low-composite score, a “vestibular” pattern on sensory organization testing (SOT), and an ankle dominant sway pattern in conditions 5–6. CDP has some utility in distinguishing malingering of imbalance (131) from BVW; however, patients with severe BVW can be falsely categorized as “aphysiologic” on SOT algorithms (132), so the result should not be interpreted in isolation. These ambiguities pose difficulties in medico-legal situations, as can arise in cases of gentamicin ototoxicity.

## Treatment for BVW

It is rare that the underlying cause of BVW can be directly treated, so it is important to recognize such cases (e.g., treatment of syphilis or AIED, stopping an aminoglycoside antibiotic). Vestibular hair cells do not seem to exhibit any regenerative capacity (133) in humans, so it is unlikely that any treatment will improve or reverse peripheral vestibular damage. Central compensation, likely mediated by plasticity of the commissural connections between the vestibular nuclei (134), appears to require that there be some minimum residual peripheral vestibular function (135) but even when this mechanism is available, improvement in the VOR is very limited (136–138), and cannot restore the VOR to its premorbid level. In clinical practice, the lack of substantial plasticity in the VOR is easily appreciated when one does vHIT testing in patients with longstanding gentamicin-induced bilateral loss. Even after many years, VOR gain remains extremely low.

Thus, recovery of the VOR in bilateral vestibular loss is based on substitution. Most BVW patients improve with physical therapy, though this must be appropriately targeted vestibular rehabilitation therapy (139). Such therapy attempts to teach patients compensatory strategies by relying more heavily on their intact sensoria (vision and proprioception) and improving their internal estimates of motion.

Some entrenched practice patterns in medicine can be problematic for BVW patients. Regrettably, many of these patients are “diagnosed” with “vertigo” and started on vestibular suppressants such as meclizine or a benzodiazepine, which will diminish the already deficient peripheral vestibular input and worsen imbalance. More insidious than this is the use of medications that have the unintended adverse effect of vestibular suppression, and care should be taken to avoid such medications. For example, when treating depression in BVW patients it would be preferable, where medically feasible, to avoid medications with anti-histaminic or anti-cholinergic effects (such as tricyclic compounds); when treating anxiety it would be preferable to avoid benzodiazepines.

Vestibular prosthetic devices (140) are being developed and tested, but remain investigational. Another approach involves technology intended to improve or restore inner ear function by coaxing human inner ear hair cells to regrow, similar to some species of birds (141, 142).

## Natural History of BVW

The more extensive the damage in BVW, the more pronounced the symptoms (143). After 1–2 years, patients with mild BVW may be indistinguishable from normal controls. Patients with moderate BVW will complain of persistent ataxia and oscillopsia. Patients with severe BVW complain not only of ataxia and oscillopsia, but also of limited function in their daily activities; for example, severe BVW patients generally refrain from driving. The presence of other relevant sensory deficits (e.g., visual impairment from macular degeneration, proprioceptive impairment from peripheral neuropathy) or motor deficits (e.g., paresis from stroke, mechanical limitations from orthopedic problems involving the spine or legs) interferes with the development of compensatory strategies, and such patients tend to have worse outcomes.

While most patients with BVW can improve to some extent, the degree of improvement depends on the underlying etiology (143, 144). In cases of gentamicin ototoxicity, the medication’s active toxicity continues for some time even after the offending agent is stopped due to some of its pharmacologic properties (see discussion above). Nevertheless, measurable improvement in the VOR begins within about 3 months and can continue for up to approximately 2 years (145). In moderate to severe BVW cases, patients rarely return to their previous level of function; on physical examination they will continue to perform poorly on the DVA (see above). Patients with mild BVW, and even some with moderate BVW, may eventually resume driving, though may still avoid driving at night. If a patient’s occupation is sedentary or at least does not rely on having good balance, they are often able to resume work.

## Mechanisms for Improvement

Maximum medical improvement is usually reached after about 2 years. The main mechanisms of improvement are central compensation, peripheral recovery, and behavioral adaptation.

Central compensation refers to the idea that the brain re-prioritizes sensory input by relying more heavily (in engineering terms, “upweighting the gain”) on non-vestibular input (e.g., vision, proprioception) (146). Data from animal studies suggest that the process of central compensation involves neural plasticity *via* new synapse formation in the vestibular nuclei of the brainstem (147). In humans, there is also evidence from fMRI studies that cortical reorganization occurs (148). Central compensation, defined as increasing the VOR gain, seems to limited to roughly a factor of 2 (149, 150). Considering that many bilateral patients have lost all or perhaps 90% of their VOR, a factor of 2 is woefully inadequate.

Evidence from animal studies suggests that vestibular hair cells that are merely damaged (but not dead) may have some capacity to recover function (151); it is plausible that this occurs in humans as well. Similarly, there is sometimes recovery of nerve function in vestibular nerve injuries due to vestibular neuritis; by 6 months after a vestibular nerve injury, any recovery that is going to occur is probably complete by approximately 6 months (144, 152).

Behavioral adaptations, whether instinctive or planned, play a role in adjusting to BVW. Patients tend to avoid activities and situations in which an unanticipated loss of equilibrium would endanger them. Most BVW patients with moderate to severe BVW avoid driving at night, riding bicycles, climbing, and standing on ladders. They are also aware of circumstances that would temporarily limit their vision (e.g., walking in a poorly illuminated area) or challenge their proprioception (e.g., walking on a rough or uneven surface). We have not personally encountered patients with BVW experiencing problems with swimming; however, swimming imposes proprioceptive and visual challenges, and there are additionally theoretical grounds (153) to suspect that swimming may be difficult for these patients.

## Conclusion

Bilateral vestibular weakness refers to reduced or absent vestibular function on both sides, and nearly always arises from disease affecting the labyrinths or vestibular nerves. Presenting symptoms are oscillopsia and imbalance. BVW is rare; the typical causes include gentamicin ototoxicity; less common causes include AIED, meningitis, and bilateral vestibular neuritis; in about half of the cases no etiology can be determined. Bedside examination techniques (DVA testing, HIT, and ophthalmoscope test) can be helpful but are not sensitive. RCT remains the gold standard for diagnosing BVW; vHIT may be a reasonable screening test, but requires further study (specifically comparing it to RCT). VNG and VEMPs play a lesser role in diagnosis. Treatment is with vestibular rehabilitation therapy, focusing on sensory substitution. Central compensation is believed to be the mechanism underlying any measurable improvement in the VOR. Clinical improvement generally plateaus at approximately 2 years.

## Author Contributions

TH came up with the main concepts for the article and wrote sections of a preliminary draft. MC and DY edited and added to the draft. MC rewrote the manuscript into its final form.

## Conflict of Interest Statement

The authors declare that the research was conducted in the absence of any commercial or financial relationships that could be construed as a potential conflict of interest.
